# From Shadows to Spotlight: Courageous Followers Shape Leaders Perceptions and Influence Followers’ Job Performance

**DOI:** 10.1155/jonm/9927655

**Published:** 2026-01-19

**Authors:** Wajeeha Brar Ghias, Hafsah Zahur

**Affiliations:** ^1^ Department of Leadership & Management Studies, National Defense University (NDU), Islamabad, Pakistan; ^2^ Faculty of Management Sciences, National University of Modern Languages (NUML), Islamabad, Pakistan

**Keywords:** courageous followership, follower performance, hierarchical culture, implicit theories, nursing leadership

## Abstract

**Background:**

In health care, leadership is often viewed through top–down lens, which leaves the active role of followers unrecognized. Nurses do not just comply with orders—they shape leadership, culture, and performance through their behavior. Courageous followership, reflected in accountability, integrity, and speaking up, is central to this influence. Yet, this perspective remains underexamined in hierarchal healthcare environments.

**Aim:**

This study examines how courageous followership affects leaders’ implicit followership theories (LIFTs) and followers’ job performance. It also explores LIFTs as mediating mechanisms within healthcare teams.

**Methods:**

Multisource data were collected from 472 healthcare professionals in Pakistan using cross‐sectional survey design. Partial least squares structural equation modeling (PLS‐SEM) was used to test direct and mediated relationships among five courageous followership dimensions, LIFTs and follower job performance.

**Results:**

Four dimensions of courageous followership, that is, assume responsibility, serve, challenge, and participate in transformation—positively influence both LIFTs and job performance with LIFTs as a mediator. However, the dimension of “taking moral action” had a negative effect, suggesting that ethical dissent may be viewed unfavorably in high‐power distance environments. The structural model demonstrated strong explanatory and predictive power (*Q* ^2^ for LIFT = 0.587 and *Q* ^2^ for performance = 0.478).

**Conclusion:**

This study offers a novel theoretical integration of followership theory and implicit followership theories, emphasizing how followers’ courage, ethics, and agency shape leadership perceptions and outcomes. It shifts attention from leader‐centric views to more relational, followers‐focused understanding of healthcare leadership.

**Implications for Nursing Management:**

Health care leaders should recognize and encourage follower behaviors that promote responsibility with accountability. Leadership development must address perceptual biases, strengthen leader openness to follower contributions to fully harness the value of nursing followership.

## 1. Introduction

In today’s competitive world, followers are no longer the silent observers standing in the shadows while leaders take the spotlight—traditional leadership is evolving with recognition of the shared role played by both leaders and followers. Leadership, tightly bundled with hierarchy, is really effective in resolving the collective issues of humanity and global challenges [1]. Particularly, during a crisis, healthcare leaders succeed only with the support of active and engaged followers [2]. Effective followership plays a transformative role in addressing burnout and enhancing both individual and organizational wellness [3]. In spite of this, followership remains a relatively underexplored area, especially in health care, where its significance is overlooked [4, 5]. Professional nursing education emphasizes assertive behavior, and critical incident reporting, only as competencies for nurses’ leadership and not as followership skill [6].

Leadership research raises a critical question considering the role of leaders in addressing societal challenges: “to what extent do follower behaviors and contextual factors shape leader behavior and its outcomes” [1]. Among various follower behaviors, courageous followership comprises five dimensions: assuming responsibility of one’s work, serving the leader and organization constructively, challenging authority, participating in organization transformation, and acting on moral principles‐even when it involves personal risks [7].

Courageous followers are present at all organizational levels, and they demonstrate high level of personal responsibility, moral courage, and commitment to organizational goals [8, 9]. In spite of its relevance, the courageous followership framework has received less scholarly focus in South Asian contexts, where Kelley’s [10] work continues to be the focus of researchers’ attention [11, 12]. The existing literature on courageous followership remains methodologically limited, with more attention on qualitative designs [4, 13, 14] or basic quantitative designs [8, 9, 15] which offer only surface‐level inquiry in the complex organizational environments [16].

Courageous followership is essential in high power‐distance societies [17] like Pakistan where nurses face barriers [18] such as limited advancements, weak administrative support, and interpersonal hostility. Nurses feel challenged to question unethical practices leading to the emergence of toxic triangles in the system [19], but keeping in view patient care and safety, nurses need to inculcate initiative taking behavior in spite of the personal risk of proactivity [11, 20]. On the other hand, the follower’s proactive behavior depends on how it is supported by workplace leadership [4, 21] since high‐quality leader–member exchange (LMX) can enable nurses to speak up for patient safety [18], underlining the crucial role of leader’s openness to enable followers’ actions. This aspect leads to another critical yet under explored area in followership research. that is. leaders’ implicit followership theories (LIFTS) [16].

In hospital settings, nurses act as the first point of action, making their voice with vigilance and moral courage to deliver better healthcare services but often positioned as passive actors who merely execute directives and can make ethical choices [4]. This gap is particularly significant in nursing, where moral courage and assertive followership are essential for patient advocacy and safety [4, 14]. Therefore, it is critical to investigate the schemas nurses’ leaders hold about courageous followership to improve healthcare teams’ effectiveness [21].

Role‐based theory of followership, reversing the lens of leadership, redefines followership as an active role rather than a subordinate role [22, 23]. The focus of theory is to put followers at the forefront and emphasize what followers do, the role they enact, and what is the impact of enactment on leadership process. Followership theory redefines followers as active agents of change that help in co‐producing leadership [7, 10, 23]. This followership approach is also complemented by LIFTs that endorse that leaders hold personal perceptions about followers’ behaviors and those assumptions impact leaders’ behavior [22, 24].

Parallel to role theory, LIFTs argue that leaders depend on mental models, formed through experience and expectations, which guide leaders to interpret followers’ performance [25]. Positive LIFTs are mental models that leaders perceive about followers as good citizens, enthusiastic, and industrious, [24]. In high power distance contexts, these subconscious evaluations may play a crucial role for courageous followership, where dissent is discouraged. Although leaders with positive LIFTs (prototypes) tend to provide high‐performance ratings to followers [26, 27] and develop liking for followers with strong leader–member exchange relationships [24, 28], how courageous followership shapes leaders’ cognition or prototypes remains unexplored.

**Figure 1:**
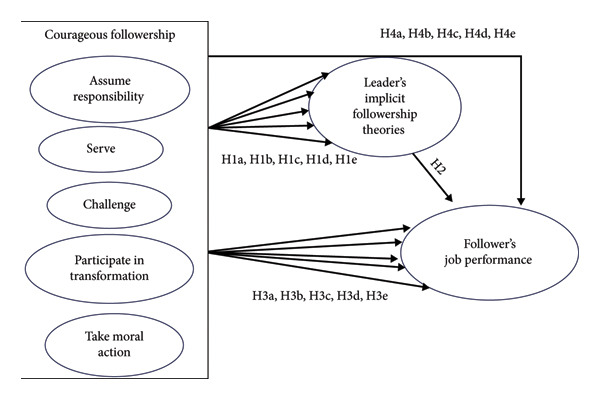
H1a H1b H1c, H1d, and H1e (direct impact of courageous followership dimensions on leaders’ implicit followership theories). H2 (direct impact of leaders’ implicit followership on followers’ performance rating). H3a H3b H3c, H3d, and H3e (direct impact of courageous followership dimensions on followers’ performance rating). H4a H4b H4c, H4d, and H4e (simple mediation path of Leaders’s Implicit Followership Theories (LIFTs’) between courageous followership dimensions and followers’ performance rating).

Given the gaps in the literature and theoretical support from followership theory, this study argues that follower’s behavior and leader cognition must be studied together for a better understanding of how it works the in healthcare setting. The role‐based theory of followership helps in framing the enactment of courageous followership, such as assuming responsibility with ethical proactivity, while LIFTs develop the framework to understand perceptual consequences of courageous followership. The proposed research model is shown in Figure 1.

Drawing on the integrated framework, this study investigates how courageous followership dimensions are linked to leader‐rated job performance among nurses, which is mediated through leaders’ positive prototypes of their followers. This study shifts the focus from leadership actions alone to synergy between follower agency and leader cognition by examining supervisor–nurse dyads. Particularly, this study is relevant in the South Asian healthcare context where followers’ behavior and actions are interpreted and rewarded in light of hierarchal dynamics and cultural norms [17].

## 2. Literature and Hypotheses

### 2.1. Courageous Followership and LIFTs

Courageous followership emphasizes the active role of followers in supporting and challenging leaders to drive organizational transformation [7, 8]. This approach closely aligns with LIFTs, which encompass ideal follower traits such as enthusiasm and good citizenship [24]. Courageous followers demonstrate commitment by setting positive examples and engaging constructively with leaders and organizational processes [4]. Such active participation and willingness to challenge the status quo exemplify the traits leaders value in their followers, including enthusiasm for change and proactivity in voicing concerns [5, 24, 27]. Courageous followers serve both the leader and the organization aligning with positive followership traits of good citizenship [9].

Courageous followers who take moral action (TMA) not only challenge wrongdoing but also serve as ethical anchors within an organization [7] are closely aligned with positive LIFTs. Leaders perceive such behavior as positive cues, a dependable proactive follower, and develop good perception [29]. The above literature and role‐based followership theory suggests that by engaging in courageous followership, individuals signal competence, integrity, and commitment in leaders’ mind, and it turns into positive prototypes [23, 24]. In line with the above theoretical reasoning, the following hypotheses are developed to examine the proposed relationships.

H1: Courageous followership, characterized by (a) assuming responsibility, (b) serving, (c) challenging, (d) participating in transformation, and (e) taking moral action, positively influences leaders’ implicit follower theories (LIFTs).

### 2.2. LIFTs and Job Performance

Leaders’ perceptions significantly influence their behavior toward subordinates [34]. Followership theories suggest that leaders possess high expectations and favor followers who are diligent and collaborative, often exceeding their performance expectations [24]. When leaders perceive followers as industrious and enthusiastic, it results in elevated performance evaluations [27]. According to Sy’s [24] theory, leaders develop schemas about their followers through interactions. Followers who engage in challenging tasks create impressions of diligence [35] leading leaders to view them as positive role models. These prototypes subsequently influence leadership behavior and result in favorable outcomes such as high‐performance ratings [27, 32], leader’s trust [24], and career success [26]. Consequently, leaders with positive implicit followership theories are inclined to rate higher followers’ performance leading to following hypothesis:

H2 Leaders’ implicit follower theories (LIFTs) positively influence followers’ job performance.

### 2.3. Courageous Followership and Followers’ Job Performance

Followers play a crucial role in the leadership process through their behavior [27, 30]. Courageous followers exhibit self‐motivation and adhere to performance standards, thereby enhancing their overall performance [31]. Engaged followers surpass expectations and offer support during changes in the organization [12]. Employees with proactive followership styles demonstrate higher job performance than passive followers [32]. Courageous followership is characterized by proactive behavior in identifying organizational issues [29]. Employees who exhibit proactivity add value to constructive feedback and usually emerge as exemplary performers [28, 33]. The role‐based followership theory posits that followers who are responsible for their own outcomes [23] can be related with courageous followers’ behavior who identify and address organizational issues [7] and considered as change agents and leaders labeled them high performers [33]. Based on this theoretical lens, such behavior can impact leader‐rated performance leading to the following hypotheses:

H3 Courageous followership (a) assuming responsibility, (b) serving, (c) challenging, (d) participating in transformation, and (e) taking moral action positively influences followers’ job performance.

### 2.4. Mediation Mechanism LIFTs

The follower role‐based approach posits that followers play a key role in influencing leaders and organizational outcomes through their upward influence [23]. Courageous followers demonstrate responsibility, articulate ethical concerns, and propose innovative ideas, thereby enhancing performance evaluation and career advancement [33], challenging established norms, offering constructive feedback, and advocating for change; such followers positively impact performance [8]. However, the outcomes of followers are contingent upon leaders’ perceptions and evaluations of followers’ behaviors [24]. Leaders value engaged and industrious followers as positive prototypes, fostering a reciprocal influence dynamic [27]. Leaders’ assumptions about followers, shaped by positive prototypes, connect followers’ behavior to performance evaluations [28]. Thus, courageous followership is anticipating enhanced job performance through positive LIFTs leading to hypothesize mediation mechanism.

H4 Leaders’′ implicit followership theories (LIFTs) will mediate the relationship between courageous followership and (a) assume responsibility, (b) serve, (c) challenge, (d) participate in transformation, and (e) TMA, and enhance followers’ job performance.

## 3. Material and Methods

### 3.1. Design and Sample

The study adopted a hypothetic‐deductive approach, considering literature gaps and contextual relevance. The target population comprised 103,039 registered nurses in Pakistan holding 4‐year degrees. Because of logistic and financial constraints, the study was delimited to major hospitals in Punjab (Rawalpindi Lahore, and Faisalabad), Khyber Pakhtunkhwa (Peshawar, Kohat, and Abbottabad) and Islamabad—representing approximately 70% of the national nursing workforce. Hospitals were selected based on the World Health Organization (WHO) criteria, including a minimum of 100,000 in‐patient beds. Prior to data collection, hospital administrations were formally contacted through official correspondence and follow‐up meetings with nursing directors to explain the study objectives, ensure institutional support, and obtain ethical clearances.

A convenience sampling strategy was employed to deal with demanding schedules of the nursing staff. Data were collected over a 6‐month period (July–December, 2024) using a paper‐pen‐based survey. We recruited a total of 600 nurses, including 100 nurse leaders and 500 junior nurses. The sample size was planned to deal with missing responses and achieve precision of PLS estimates [36, 37]. The multisource data were collected from two professional roles, to rationalize follower‐level outcome, that is, follower behavior and leader cognition. Junior nurses rated their courageous followership, and nurses leaders rated junior nurses’ implicit followership and performance rating. The 1:5 ratio between nurse leaders and followers reflects common staffing pattern in Pakistani hospitals [38] In addition, nurse leaders with a narrow span of control can better asses their followers [39]. Each junior nurse was linked, coded to their immediate leader, and data nested into the junior nurse record, forming 500 leader–follower dyads. Using multisource dyadic data minimizes the chance of common method bias [40]. After matching responses and removing incomplete data, 390 followers matched with 82 leaders were retained.

### 3.2. Instruments

All constructs were measured using a five‐point Likert scale (1 = strongly disagree, 5 = strongly agree). Courageous followership was measured using the 19‐item Gene Dixon’s [31] scale asking participants to describe themselves on five dimensions as: assume responsibility (five items, α = 0.839–sample item “*passionate about my work and commitments,”* serve the leader (five items, α = 0.833)–sample item “*minimizes unnecessary pressure on the manager, ” and* challenge the leader (two items, α = 0.711)–sample item “*confronts group thinking.”* The fourth dimension, participate in transformation (four items, α = 0.869)–sample item “*provides support for experimentation and learning”* and TMA (three items, α = 0.873)–sample item “*would resign to protect my manager from repercussions.”* LIFTs were measured using Thomas Sy’s [24] nine‐item scale (α = 0.912)–sample item “My subordinate is hardworking.*”* Follower job performance was rated by leaders using the seven‐items scale developed by William and Anderson’s [41] (α = 0.883)–sample item “*My subordinate meets formal performance requirements of the job.”*

### 3.3. Assessment Method

Structural equation modeling (SEM) is used to estimate complex models with multiple dimensions and variables [36, 42]. This study opted for SmartPLS4 [43] to analyze the proposed research model depicted in Figure 1. PLS‐SEM techniques test the relationships between multiple observed and latent variables in a single framework, for estimation and accuracy of predictive performance of model, with recent development in machine learning to improve predictive accuracy (Q2) and reveal hidden patterns [36, 37].

## 4. Results

### 4.1. Sample Characteristic and Descriptive Statistics

To better understand the context of the study population, the demographic characteristics of the respondents are summarized. In terms of designation, out of the total 472 nurses, junior nurses constitute *n* = 390, while senior nurses who reported as source of data constitute n = 82. Female participants dominated the gender proportion (*n* = 417, 88%), with male participants accounting for only 12% (*n* = 55). Majority of the participants were between 20 and 25 years of age (*n* = 252, 55%), followed by 26–30 years (*n* = 138, 29%), 31–35 years (*n* = 52, 11%), 36–40 years (*n* = 19, 4%), and those aged 40 years and above (*n* = 11, 2%). The tenure of the nurse leader is also varied with 2.5–3 years (*n* = 209, 54%), 3.5–4 years (*n* = 72, 18%), 4 years or more (*n* = 52, 15%), and 1–2.5 years (*n* = 57, 15%).

To conduct complex model assessment, raw data need to be analyzed for initial descriptive insights and baseline understanding of the model. The results of Table 1 show all variables’ moderate average score ranging from 3.3 to 3.7 with S.D. ranging from 0.94 to 1.3, which constitutes moderate to high variability.

**Table 1:** Descriptive statistics and correlation matrix (*n* = 390).

Variables	M	Sd	AR	CH	FJP	LIFT	PIT	SR	TMA
AR	3.5	0.97	—						
CH	3.3	1.1	0.42^∗∗^	—					
FJP	3.7	0.95	0.55^∗∗^	0.50^∗∗^	—				
LIFT	3.6	0.94	0.61^∗∗^	0.53^∗∗^	0.62^∗∗^	—			
PIT	3.4	1.2	0.46^∗∗^	0.39^∗∗^	0.53^∗∗^	0.58^∗∗^	—		
SR	3.5	1.0	0.49^∗∗^	0.39^∗∗^	0.51^∗∗^	0.58^∗∗^	0.41^∗∗^		
TMA	2.6	1.3	−0.45^∗∗^	−0.33^∗∗^	−0.47^∗∗^	−0.51^∗∗^	−0.37^∗∗^	−0.411^∗∗^	—

*Note:*​ Serve (SR), Challenge (CH), M (Mean).

Abbreviations: AR, Assume Responsibility; FJP, Followers′ Job Performance; LIFT, Leaders′ Implicit Followership Theories; PIT, Participate in Transformation; S.D, Standard Deviation; TMA, Take Moral Action.

^∗∗^Significance Level, *p* ≤ 0.01.

The correlation matrix shows that four courageous followership dimensions (AR, CH, SR, and PIT) have strong positive association with leaders’ perceptions and performance rating (Table 1). The fifth dimension (TMA) has negative correlation with constructs (*r* = −0.33 to −0.51. *p* < 0.1).

### 4.2. Measurement Model Assessment

To ensure the robustness of the structural model, both collinearity and common method bias were systematically examined. Item‐level collinearity was assessed using variance inflation factor (VIF), with all values falling well below the recommended threshold [37] of 3.3, ranging from 1.453 to 2.774, confirming that multicollinearity among the indicators is not an issue. Full collinearity VIF values of all constructs (Table 2) were also below the critical threshold of 3.3, ranging from 1.042 to 2.075; common method bias is not prevalent [44].

**Table 2:** Measurement Model Assessment (Reliability & Discriminant validity).

Construct	CMB	Reliability		HTMT ratios and Fornell–Larker criterion						
	FVIF	α	*ρ*_c	AR	CH	FJP	LIFT	PIT	SR	TMA
AR	1.733	0.839	0.886	**0.782** ^**∗**^	0.424	0.554	0.610	0.466	0.497	−0.452
CH	1.428	0.717	0.876	*0.545*	**0.883** ^**∗**^	0.501	0.535	0.399	0.391	−0.334
FJP	2.075	0.912	0.928	*0.642*	*0.629*	**0.768** ^**∗**^	0.628	0.536	0.513	−0.470
LIFT	2.075	0.912	0.928	*0.695*	*0.661*	*0.697*	**0.766** ^**∗**^	0.586	0.581	−0.516
PIT	1.551	0.869	0.911	*0.545*	*0.504*	*0.611*	*0.657*	**0.848** ^**∗**^	0.415	−0.379
SR	1.042	0.833	0.882	*0.594*	*0.515*	*0.596*	*0.642*	*0.490*	**0.775** ^**∗**^	−0.411
TMA	1.411	0.873	0.925	*0.527*	*0.421*	*0.533*	*0.575*	*0.432*	*0.481*	**0.896** ^**∗**^

*Note:* Full Collinearity Variance Inflation Factor (FVIF), Common Method Bias (CMB), Cronbach’s alpha (α), Composite Reliability (*ρ*_c), ^**∗**^Values below the diagonal (in italics) represent the HTMT ratios. Values on and above the diagonal represent the Fornell–Larcker criterion, with the diagonal (bold) indicating the square root of the AVE.

**Figure 2:**
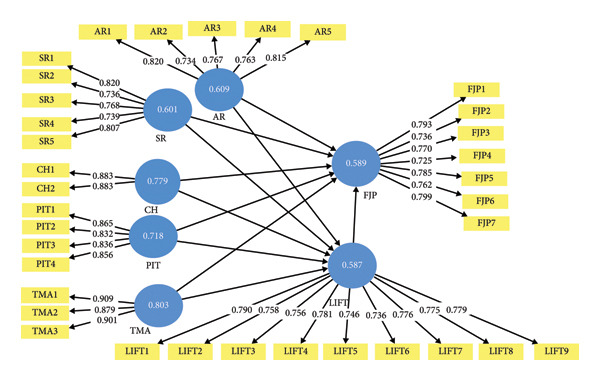
Convergent validity (outer loading and average variance extracted (AVE)).

The measurement model validated strong psychometric properties (Table 2). All constructs were within recommended thresholds for internal consistency, with Cronbach’s alpha (α) and composite reliability (*ρ*_c) between 0.876 and 0.928, which confirms measurement reliability. Convergent validity was also proven (Figure 2), as average variance extracted (AVE) values fall above the 0.50 cutoff [45], ranging from 0.587 (LIFT) to 0.803 (TMA). Indicator reliability was also confirmed, as most of the outer loadings (Figure 2) exceeded the recommended threshold of 0.70, indicating satisfactory item convergence. Across constructs, item loadings consistently ranged between 0.725 and 0.909, affirming the robustness of the reflective measurement model.

Discriminant validity was assessed using two complementary approaches: the Heterotrait–Monotrait (HTMT) ratio of correlations and the Fornell–Larker criterion. As shown in Table 2, the HTMT ratios (reported below the diagonal) were all below the conservative threshold of 0.85, as recommended by Henseler [42]. The Fornell–Larcker criterion [45] further confirmed discriminant validity, as the square root of the AVE for each construct exceeded its correlation with the other constructs (Table 2). For example, CH (0.883) and TMA (0.896) showed higher AVE square roots than their respective inter‐construct correlations. Combined with the HTMT results, the findings affirm that all the constructs are conceptually and statistically distinct.

The validity of the partial least squares (PLS) model was further assessed using the global goodness‐of‐fit (GoF) criterion. Global GoF evaluates the validity of the PLS model and assesses the effectiveness of the research model by averaging the average variance extracted (AVE) and the mean R^2^ of the variables under investigation [46]. A GoF threshold value < 0.15 suggests poor fit, whereas GoF value > 0.365 confirms good fit. The overall model’s PLS validity was established having value GoF = 0.60. Finally, model fit assessment was observed before analyzing the structural relationship among variables. The results of model fit indices are within the threshold values ([36], SRMR = 0.051, d_ ULS = 1.656, d_ *G* = 0.749, and NFI = 0.791. The comparison of saturated and estimated model fit indices showed identical values of unweighted least square discrepancy (d_ ULS) and geodesic discrepancy (d_ *G*) confirming that structural constraints imposed on the model did not reduce model fit.

### 4.3. Structural Model Assessment

**Figure 3:**
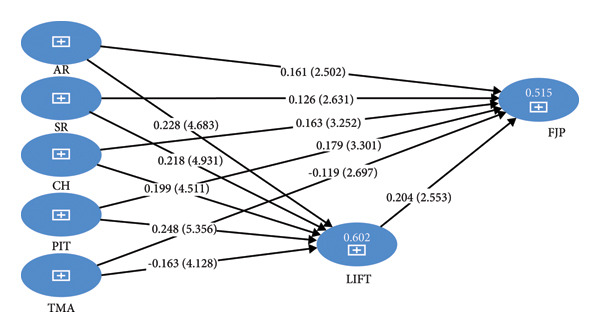
Path analysis bootstrapping results.

Bootstrapping, a nonparametric resampling technique, was employed using 5000 subsamples to rigorously assess the stability and significance of the path coefficients of the structural model in Smart PLS 4 [43]. A two‐tailed test was applied to ensure unbiased estimates of statistical significance and to accurately calculate standard errors, statistics, and *p* values for evaluating hypothesized relationships among constructs within the structural model. The results in Table 3 and path analysis Figure 3 provide substantial empirical support for all hypothesized relationships.

**Table 3:** Direct effects of courageous followership dimensions on LIFTs and FJP.

Hypotheses	Direct path	β	Sd	*t* value	CI 95%		Decision
					LB	UB	
H1a	AR ‐> LIFT	0.228	0.049	4.683	0.133	0.329	Accept^∗^
H1b	SR ‐> LIFT	0.218	0.044	4.931	0.134	0.305	Accept^∗^
H1c	CH ‐> LIFT	0.199	0.044	4.511	0.113	0.286	Accept^∗^
H1d	PIT ‐> LIFT	0.248	0.046	5.356	0.158	0.339	Accept^∗^
H1e	TMA ‐> LIFT	−0.163	0.039	4.128	−0.238	−0.091	Reject^∗^ (Opposite)
H2	LIFT ‐> FJP	0.204	0.080	2.553	0.046	0.356	Accept^∗^
H3a	AR ‐> FJP	0.161	0.064	2.502	0.039	0289	Accept^∗^
H3b	SR ‐> FJP	0.126	0.048	2.631	0.039	0.228	Accept^∗^
H3c	CH ‐> FJP	0.163	0.050	3.252	0.065	0.263	Accept^∗^
H3d	PIT ‐> FJP	0.179	0.054	3.301	0.077	0.287	Accept^∗^
H3e	TMA ‐> FJP	−0.119	0.044	2.697	−0.207	−0.045	Reject^∗^ (Opposite)

*Note:* Significance Level, ^∗^ *p* ≤ 0.05.

Abbreviations: CI = Confidence Interval, LB = Lower Bound, UB = Upper Bound.

Four dimensions of courageous followership–assume responsibility (AR), serve (SR), challenge (CH), and participate in transformation (PIT)–exhibited significant and positive effects on LIFTs with standardized path coefficients (*β*) ranging from 0.19 to 20.24 and *t* values well above the 1.96 threshold (e.g., PIT ‐> LIFT: *β* = 0.248, *t* = 5.35, 95% CI [0.158–0.339], *p* < 0.001), thereby supporting hypotheses H1a through H1d. Conversely TMA demonstrated a significant negative impact on LIFT *β* = −0.163, *t* = 4.12, 95% CI [−0.23, −0.91] *p* < 0.001), contradicting the hypothesized positive direction and leading to the rejection of hypothesis H1e.

In line with H2, LIFTs significantly and positively predicted followers’ job performance (*β* = 0.204, *t* = 2.553, 95%CI [0.046, 0.356]), indicating that leaders’ perceptions of ideal followers were associated with improved job performance ratings. Furthermore, AR, SR, CH, and PIT had a significant positive effect on FJP (*β* = 0.126–0.179, *t* > 2.50) and all confidence intervals do not include zero (Table 3), providing support for hypotheses H3a through H3d. However, similar to its effect on LIFTs, TMA showed a significant negative relationship with FJP (*β* = −0.119, *t* = 2.697, 95%CI [−0.207, −0.045], *p* < 0.01), leading to the rejection of H3e.

Collectively, these findings of direct path analysis suggest that most dimensions of courageous followership positively contribute to leaders’ perceptions and job performance ratings. However, moral action when exercised independently by followers may be perceived as misaligned or disruptive in certain organizational contexts, resulting in adverse performance rating outcomes.

Table 4 Presents the indirect effects assessed by bootstrapping. The analysis revealed that leader’s implicit followership theories (LIFTs) significantly mediated the relationship between four courageous followership dimensions (AR, SR, CH, and PIT) and follower job performance (FJP), with positive indirect effects (*β* ranging from 0.040 to 0.0500); all *t* values > 1.96, *p* < 0.05, and all confidence intervals do not include zero. Therefore, hypotheses H4a–H4d are accepted. Conversely, the indirect effect involving take moral action (TMA) was significantly negative (*β* = −0.033; *t* = 2.080, 95% CI [−0.068, −0.006]), leading to the rejection of H4e, as it contradicted the hypothesized positive direction.

**Table 4:** Specific Indirect Effects of Courageous followership Dimensions on FJP via LIFTs.

Hypotheses	Indirect path	β	Sd	*t* value	CI 95%		Decision
					LB	UB	
H4a	AR‐> LIFT ‐> FJP	0.047	0.019	2.505	0.011	0.083	Accept^∗^
H4b	SR ‐> LIFT ‐> FJP	0.044	0.019	2.287	0.009	0.085	Accept^∗^
H4c	CH ‐> LIFT ‐> FJP	0.040	0.019	2.182	0.008	0.080	Accept^∗^
H4d	PIT ‐> LIFT ‐> FJP	0.050	0.021	2.351	0.011	0.095	Accept^∗^
H4e	TMA ‐> LIFT ‐> FJP	−0.033	0.016	2.080	−0.068	−0.006	Reject^∗^(Opposite)

Abbreviations: CI = Confidence Interval, LB = Lower Bound, UB = Upper Bound.

^∗^ *p* value Significance Level, *p* ≤ 0.05.

### 4.4. Model Predictive Strength and Explanatory Power

The coefficient of determination (*R* ^2^) values for LIFTs (0.603) and FJP (0.516) indicate that the structural model accounts for a substantial proportion of the variance in both endogenous constructs (Table 5). Cohen’s effect sizes [47] help in establishing practical significance of the study. LIFT and all dimensions of courageous followership demonstrated small to moderate effect sizes representing a meaningful role in shaping leaders’ perceptions of followers’ behavior. In contrast, the predictors of FJP, including LFT, PIT, AR, CHF, TMA, and serve, exhibited small or negligible effect size (Table 5).

**Table 5:** Summary of Predictive Strength and Explanatory Power (*R* ^2^, *f* ^2^. *Q* ^2^).

Endogenous constructs		Values		Path	*f* ^2^	*t* value	*P* values
Leader’s implicit followership theories				AR ‐> LIFT	0.080	2.196	0.028^∗^
*R* ^2^	Adj *R* ^2^	0.602^∗∗∗^	0.597^∗^	SR ‐> LIFT	0.079	2.283	0.022^∗^
*Q* ^2^ predict		0.586		CH ‐> LIFT	0.073	2.206	0.027^∗^
RAMSE	MAE	0.646	0.483	PIT ‐> LIFT	0.107	2.390	0.017^∗∗^
				TMA ‐> LIFT	0.048	1.985	0.047^∗^
				LIFT ‐> FJP	0.034	1.094	0.274
Followers job performance				AR ‐> FJP	0.030	1.109	0.268
*R* ^2^	Adj *R* ^2^	0.515^∗∗∗^	0.509^∗^	SR ‐> FJP	0.020	1.175	0.240
*Q* ^2^ predict		0.478		CH ‐> FJP	0.037	1.512	0.131
RAMSE	MAE	0.725	0.519	PIT ‐> FJP	0.041	1.561	0.119
				TMA ‐> FJP	0.020	1.279	0.201

Abbreviations: MAE, Mean Absolute Error; RMSE, Root Mean Square Error.

^∗^ *p* ≤ 0.05.

^∗∗^ *p* ≤ 0.01.

^∗∗∗^ *p* value Significance Level *p* ≤ 0.001.

Although FJP shows a moderate *R* ^2^ = 0.516, the very small *f* ^2^ value (0.034) indicates that no single predictor on its own can explain this variance. It is not a contradiction; *R* ^2^ reflects combined influence of all predictors, while *f* ^2^ captures the unique impact of each variable. The small effect size of LIFTs suggests that it has limited direct effect on follower job performance, yet it is still meaning to overall model. The result indicates that LIFTs impact job performance more through indirect pathways rather than through direct link. This interpretation is consistent with implicit followership theoretical assumptions that it works for influencing cognition, a view further supported by significant indirect effects identified in bootstrapping mediation results [24, 36].

The model predictive power was calculated through *Q* ^2^_predict. In Table 5, *Q* ^2^ values for LIFTs (0.586 > 0) and (0.478 > 0) exceeded the recommended threshold, confirming model accurate predictive power [36]. In addition, the Root Mean Square Error (RMSE) and Mean Absolute Error (MAE) values for the LIFTs and FJP were within acceptable limits [37]. The MAE values, both less than 0.52, indicate a minor average prediction error, reinforcing the model’s practical utility in applied settings. The superior predictive accuracy for LIFTs, as indicated by its lower RMSE and MAE and high Q2_predict, implies that leader perceptions are forecasted more reliably than follower job performance. Collectively, these findings highlight that the model exhibits moderate‐to‐strong predictive performance, with strength in predicting psychological perceptions (LIFTs) compared with more complex behavioral outcomes (FJP).

## 5. Discussion

The present study adds novel clarity to how follower behavior shapes leadership process and followers’ outcomes in health care, where empirical evidence has been limited [27]. This research confirms that followers play an active role in shaping the way leaders form judgments and how it unfolds as followers’ performance outcomes. The study findings further complement the relational view of leadership, for example, courageous followers influence leaders’ interpretations and expectations rather one‐directional guidance from the top [22, 48].

Four dimensions of courageous followership, AR, SR, CH, and PIT, showed consistent positive effects on both LIFTs and followers’ job performance, aligning with theoretical models that posit followers as proactive contributors [33, 34], which further strengthen the followership role theory view point that followers should be made responsible for followers’ outcomes [23]. Particularly, the results of AR and PIT exhibited moderate effect sizes (*f* ^2^ = 0.80 and 0.107, respectively) confirming that when followers step forward, engage in organizational improvements, and respectfully challenge, it shifts the leader cognition in favorable follower performance assessments, supporting the direct contribution of follower agency to how leaders understand and reward [24].

A notable and complex finding concerns the dimension of moral action. In spite of its theoretical alignment with ethical courage, moral action has negatively influenced relationship with both LIFTs and performance, suggesting that context plays a key role as to how ethically assertive behavior is perceived [18]. In the healthcare environment, especially in the South Asian context, speaking up on moral grounds may be interpreted as challenging authority rather than upholding ethical standards [17]. From a cross‐cultural research perspective, this study clarified that follower courage should be assessed through sociocultural norms, follower intended principled action in Asian context man be received as resistance, particularly when leaders are unaccustomed to upward voice [29].

The mediation results further clarify the underlying mechanism. LIFTs act as a cognitive bridge between follower behavior and job performance such as leaders’ perceptions that function as cognitive filters through which follower behaviors are evaluated and translated into outcomes [25, 27, 28]. This means that followers’ actions themselves do not fully determine followers’ outcomes; rather, leaders’ cognition plays a crucial role as to how leaders perceive courageous followership within hierarchical structures [33].

Taken together, the results give a deeper understanding of situating courageous followership in the sociocultural environment of high‐power distance settings. The behavior of taking responsibility and constructive challenge may be valued universally, but moral action may require cultural reframing before expressing or being interrupted. In a high‐power distance context, courageous followership cannot rely solely on individual initiatives; rather, it must be supported by leadership openness and organizational culture that value such behavior.

### 5.1. Theoretical Contribution

This study contributes to theoretical development of leadership and followership in several ways. First, it offers empirical support to courageous followership as a multidimensional construct while extending Chaleff’s framework into healthcare settings [7], confirming that proactivity influences leader cognition in a meaningful way. Secondly, the study advances implicit followership theory by confirming that cognitive prototypes shift in response to follower behaviors, validating the reciprocal nature of leadership [16, 24]. Thirdly, the findings add value to the sociocultural context. Courageous followership does not function uniformly across cultures specifically. Negative association of moral action and LIFTs reflects how norms of obedience shapes leaders’ interpretation of followers’ courage [4]. Finally, this study enhances process‐level theory by empirically demonstrating that LIFTs are critical cognitive mechanisms, rather than merely simple behavioral models, and serve as relational processes through which followers influence leadership [3, 16, 17].

### 5.2. Practical Implications

Practically, the results provide useful guidance for healthcare leaders and followers. From followership lens, organization must design followership training programs to develop followers, how to express courage in way that are tactful, culturally sensitive, and aligned with organizational norms [19]. Followers must be trained so that their performance is not shaped by leadership alone—followers must show courage to offer input and engage constructively [7]. From a leadership lens, key implication is relevant to dominance of LIFTs indirect effect, highlighting the value of leader development programs to train leaders how to interpret followers’ cues, and accept constructive criticism [17]. Leaders need to inspire and reward courage, and create a psychological safe environment where followers can speak without fear of negative consequences [33]. Finally, the performance review process should be redesigned to appreciate follower initiatives, not only focusing on task completion, yielding a more comprehensive evaluation [3].

### 5.3. Limitations and Future Research

This study has few limitations. Its cross‐sectional design restricts causal claims, highlighting the need for longitudinal or experimental designs. Reliance on leader reported performance may also create bias; future studies should use peer rating for followers’ evaluation [40]. While Pakistani nurses provide a valuable cultural perspective, generalizability remains limited, calling for cross‐cultural research. Future qualitative inquiry [14, 16] could deepen our understanding of how leaders interpret follower behaviors, including moral action, particularly within sensitive or hierarchical health environments.

## 6. Conclusion

This study brings followers from shadows to the spotlight, showing that courageous followership plays a meaningful role in shaping how leaders think and act [1]. When followers take responsibility, offer support, challenge, and transform, they influence not only their leaders, rather their own effectiveness too. Yet, the negative impact of moral courage, underlines the cultural and hierarchal role in perceptions. Improving healthcare performance requires nurturing ethically courageous followers and leaders with positive prototypes who listen to their followers. Organizations need to inculcate followership development for ethical and effective teamwork in healthcare organizations [14].

## Ethics Statement

This article is part of a PhD research project approved by the Institutional Review Board (IRB) of the National University of Modern Languages NUML‐(Ref. No. 7/MS/2024) on February 20, 2024. All procedures followed ethical standards of the Helsinki Declaration. Informed consent was obtained from all participants prior to data collection. The first author completed certified training in human subjects’ research at Albert Einstein College of Medicine on June 8, 2024, earning six interprofessional continuing education (IPCE) credits.

## Conflicts of Interest

The authors declare no conflicts of interest.

## Author Contributions

Ms. Wajeeha Brar Ghias conceived and designed the study as part of her PhD dissertation, conducted the data collection, analysis, interpretation and drafted the manuscript. Dr. Hafsah Zahur provided supervisory guidance, methodological oversight and critical review of manuscript.

## Funding

The study did not receive any funding for research.

## Data Availability

The data will be available on request from the corresponding author. The data are not publicly available because of privacy requirements of participants.
